# Traumatic stress recruits an excitatory orbitofrontal–amygdala pathway to drive maladaptive aggression

**DOI:** 10.1038/s41386-026-02432-z

**Published:** 2026-05-06

**Authors:** Mikaela L. Aholt, Jessica T. Jacobs, Magdalene P. Adjei, Nooshin Mojahed, Elana Qasem, Sandria W. Athul, Buffy Ellsworth, Jacob C. Nordman

**Affiliations:** Department of Biomedical Sciences, Division of Molecular and Integrative Physiology, Southern Illinois University School of Medicine, Carbondale, IL USA

**Keywords:** Prefrontal cortex, Stress and resilience

## Abstract

Traumatic stress is a reliable predictor of heightened aggression, yet the mechanisms linking stress exposure to aggression remain poorly understood. We previously found that traumatic stress activates the posterior ventral segment of the medial amygdala (MeApv) to drive persistent increases in aggressive behavior. However, why traumatic stress would engage an aggression-promoting pathway is unclear. To address this, we mapped the inputs to the MeApv and identified a population of excitatory, but not inhibitory, neurons in Layer 5 of the medial orbitofrontal cortex (mOFC). The OFC is classically viewed as an inhibitory brake on amygdala-driven impulses, yet emerging evidence suggests it can also facilitate context-dependent emotional responses. Notably, traumatic stress is known to disrupt OFC function and its connectivity with the amygdala, implicating this circuit in pathological aggression. Using fiber photometry, we found that traumatic stress selectively activates these MeApv-projecting excitatory mOFC (mOFC^MeApv^) neurons as well as their downstream MeApv outputs. Chemogenetic inhibition of the excitatory mOFC^MeApv^ neurons during traumatic stress exposure prevented the subsequent increase in aggression while preserving non-aggressive social behavior. Together, these findings identify an excitatory cortical–amygdala pathway that is necessary for stress-induced aggression and support a model in which traumatic stress recruits, rather than suppresses, OFC output to drive maladaptive behavior.

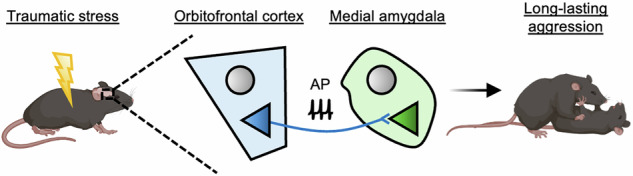

## Introduction

Pathological aggression is a prominent feature of several psychiatric disorders—including post-traumatic stress disorder, borderline personality disorder, and intermittent explosive disorder—and imposes a profound burden on patients, caregivers, and society. Critically, these aggressive tendencies often emerge after adverse experiences. Traumatic stress, in particular, produces long-lasting behavioral and neural adaptations that heighten aggression, anxiety, and depression [1–3]. Epidemiological studies underscore this link: up to 38% of violent offenders report childhood physical abuse, and exposure to early adversity increases the likelihood of intimate-partner violence more than two-fold [4–7]. Despite its prevalence, the neural mechanisms through which traumatic stress reshapes brain circuits to promote maladaptive aggression remain incompletely understood.

Previous work from our laboratory has shown that traumatic stress enhances aggression through the activation and potentiation of a pathway composed of the posterior ventral segment of the medial amygdala (MeApv) and its downstream target, the ventrolateral segment of the ventromedial hypothalamus (VmHvl)—a subcortical pathway that governs aggression, social behavior, and decision making (Fig. S1) [8–13]. Inhibiting this pathway or reversing its potentiation suppresses the aggression increase, whereas activating it during or potentiating it after stress mimics the behavioral phenotype [13–15]. However, it is not known what causes traumatic stress to recruit an aggression-driving subcortical circuit. This gap suggests that upstream regions involved in emotional regulation or behavioral control may become dysregulated by stress, thereby biasing downstream circuits toward aggression responding.

A compelling candidate for this top-down control is the orbitofrontal cortex (OFC). Traditionally studied for its roles in decision-making, emotional regulation, and reward learning, the OFC has long been considered a cortical “brake” on impulsive and aggressive behavior. Classic lesion and neuroimaging studies—from the case of Phineas Gage to modern structural and functional MRI work—identify the OFC as a critical node for emotional restraint [11, 16–29]. However, emerging evidence suggests that the OFC is not exclusively inhibitory but instead facilitates flexible, context-dependent emotional responses [17, 22, 30–32].

Interestingly, traumatic stress has been found to disrupt OFC–amygdala connectivity, leading to altered neural excitability, dendritic remodeling, and neurotransmitter imbalance [10, 17, 22, 33–36]. Stress-related reductions in OFC gray matter volume and impaired functional coupling with limbic regions have been observed in individuals with heightened impulsivity and violence [11, 16, 37]. paralleling animal evidence that stress-induced OFC dysfunction compromises behavioral inhibition [18]. Yet it remains unclear whether the OFC merely fails to inhibit aggression centers or instead aberrantly drives their activation after trauma.

Here, we test the hypothesis that traumatic stress engages a population of Layer 5 excitatory neurons in the medial orbitofrontal cortex (mOFC) that project to the MeApv (mOFC^MeApv^) to facilitate maladaptive aggression. Using fiber photometry, we recorded calcium activity from these mOFC^MeApv^ neurons and the MeApv neurons that receive mOFC input during acute foot-shock. We then chemogenetically inhibited the mOFC^MeApv^ neurons during stress exposure to test its role in subsequent aggression. Together, these experiments demonstrate that this cortical–amygdala pathway is necessary for the emergence of long-lasting aggressive behavior after traumatic stress, supporting a reinterpretation of OFC function in aggression circuits and challenging the canonical view that OFC inputs primarily inhibit amygdala-driven aggression.

## Methods and materials

### Animals

All animal protocols were approved by the Animal Care and Use Committee of Southern Illinois University School of Medicine. Wild type C57BL/6 (Charles River) and vGAT-Cre (B6J.129S6(FVB)-Slc32a1tm2(cre)Lowl/MwarJ, Jackson Labs) mice were group-housed until 4-to-5-weeks of age and then socially isolated in a 19.37 × 18.06  × 39.83 cm polycarbonate cage (Allentown) on a reverse 12-h light cycle (lights off 8:30 am–8:30 pm) with *ad libitum* access to water and food.

Surgical procedures, outlined below, were performed at 4-to-7-weeks of age, at which point the mice were single housed for an additional 3-to-4-weeks before behavioral testing. Four-to-five-week-old group-housed C57 conspecifics were used for all aggression testing. Unless noted, experiments were only performed in male mice.

### Traumatic stress-induced aggression paradigm

Single housed 8-to-11-week-old mice (experimental animals) were transferred to a darkened behavior room and allowed to acclimate for at least one hour prior to foot shock administration, as described previously [13, 15, 38, 39]. In brief, mice were placed into a shock box and after a 30 min exploration period, 15 electric foot shocks (0.5 mA, 1 s in duration) were administered through an electrified grate every 6 minutes over 90 min. Mice were then returned to their home cages. Control animals were handled identically and placed in the shock box for the same duration but did not receive foot shocks.

Seven days later, mice were transferred to the darkened room, allowed to acclimate for at least one hour, and then tested for aggression. Mice were placed into a high-walled, novel cage and left to acclimate for an additional 30 min. A neutral cage was used to minimize territorial influences on attack behavior [40]. thereby allowing us to specifically measure stress-induced aggression. Consistent with this framework, we previously reported that neither social isolation or foot shock alone reliably increases aggression in this paradigm; both are required to produce the long-lasting aggression phenotype observed in the novel cage assay [13–15, 38, 39].

Novel, group-housed conspecific mice were then placed into the arena, and the two animals were allowed to freely interact for 10 min. Conspecific stimulus mice were 4-to-5 weeks younger than the experimental animals to minimize counter-aggression and ensure that aggressive interactions were initiated primarily by the experimental mouse. Animal behavior was captured with a video camera.

Trials were monitored for excessive tissue damage (defined as open wounds or active bleeding). No such events occurred during testing, and, therefore, no trials were prematurely terminated or excluded based on injury criteria. Animals that did not display aggressive behavior were retained in the analysis to avoid biasing the dataset.

Videos of behavioral tests were reviewed and hand scored by a researcher blind to the experimental conditions. Aggressive behaviors were operationally defined as bites to the rear or face/neck/belly, boxing, chasing, and wrestling [13–15, 38, 39, 41–44]. Non-aggressive social behavior was operationally defined as anogenital sniffing, investigation, or flank rubbing. Aggression and non-aggressive social interaction were measured as percent of total time interacting, the number of interaction bouts, the duration of an individual bout, and latency to the first bout. Inter-rater reliability was confirmed using a sample dataset ( >90%). A subset of videos were rescored by the same experimenter to confirm scoring consistency (intra-rater reliability >90%). Animals with off-target viral expression were excluded from analysis regardless of behavioral outcomes.

### Stereotaxic surgery

All procedures were performed on 4-to-7-week-old male mice that were anesthetized using isoflurane (5% for induction and 1.5–2.5% for maintenance), then placed onto a stereotaxic frame (David Kopf Instruments) for viral injection and fiber implantation (Fig. S2) [43]. All viruses (Table S1) were injected using a 5 μL gas-tight Neuros-series Hamilton Syringe coupled to a 33-gauge stainless steel needle and ejected at a rate of 40 nL/min by a syringe pump (World Precision Instruments). After injection, the syringe was left in place for an additional 10 min and then slowly withdrawn. Following surgery, all mice were placed on a preheated pad to ensure core body temperature maintenance of 37 °C. Mice were then socially isolated and allowed to recover for 3-to-4-weeks before behavior testing.

For retrograde mapping of medial OFC (mOFC) projections to the MeApv (mOFC^MeApv^), mice were unilaterally injected with 300 nL of retro-pAAV-hSyn-EGFP, retro-pAAV-hSyn-DIO-EGFP, or retro-pAAV-CaMKIIα-EGFP. For anterograde mapping of the mOFC^MeApv^ pathway, mice were unilaterally injected with 300 nL of pENN-AAV1-hSyn-Cre-WPRE-hGH (AAV1-Cre) and 100 nL of pAAV9-CaMKIIα-EGFP into the mOFC and 300 nL of pAAV9-hSyn-DIO-mcherry (DIO-mCherry) into the MeApv. Skin was then sealed using Vetbond.

For single-channel fiber photometry recordings, we unilaterally injected the MeApv (AP, −1.5 mm; ML, +/−2.1 mm; DV, −5.25 mm) with 100 nL of pAAV9-CaMKIIα-mCherry and 300 nL of the following viruses: retro-pGP-AAV-Syn-FLEX-jGCaMP8m-WPRE (retro-FLEX-GCaMP8m), retro-pAAV-CaMKIIα-jGCaMP8f-WPRE, or retro-pAAV-CaMKIIα-EGFP. A black 1.25 mm diameter fiber optic ferrule was then implanted 0.1 mm above the mOFC (AP, 2.34 mm; ML, +/−0.25 mm; DV, −2.3 mm).

For dual-channel fiber photometry recordings, we unilaterally injected (i) 300 nL of AAV1-Cre and 300 nL AAV9-CaMKIIα-GCaMP6f-WPRE-SV40 or 100 nL of pAAV9-CaMKIIα-EGFP into the mOFC, and (ii) 300 nL of pAAV1-syn-Flex-NES-jRGECO1a-WPRE-SV40 or pAAV9-hSyn-DIO-mcherry into the MeApv. A black 1.25 mm diameter fiber optic ferrule was then implanted 0.15 mm above the MeApv. All fibers were secured to the skull with two stainless steel screws (1.6 mm, Protech international), a thin layer of Metabond (Parkell), and a large layer of dental cement (Lang Dental).

For chemogenetic experiments, mice were bilaterally or unilaterally injected with the following viruses: 300 nL of pENN-AAVrg-CaMKII 0.4-Cre-SV40, 300 nL of pAAV9-CaMKIIα-hM4Di-mcherry, 100 nL of pAAV9-hSyn-EGFP, and 300 nL of pAAV9-hSyn-DIO-hM4Di-mCherry, DIO-mCherry, or 100 nL of pAAV9-CaMKIIα-mCherry. Skin was then sealed using Vetbond.

### Immunohistochemistry and viral confirmation

Mice were transcardially perfused with 4% paraformaldehyde in phosphate buffered saline solution (PBS). Brains were removed and post-fixed at 4 °C overnight, then cryoprotected overnight in 15% sucrose (in PBS) followed by 30% sucrose (in PBS). Brains were cut into 30 μm thick (for antibody staining) or 40 μm thick (to confirm viral targeting) sections using a cryostat (Leica CM3050-S) and stored in PBS as free-floating sections until use. Virally injected animals were sacrificed within two weeks of the end of the experiment.

For immunohistochemistry experiments, tissue was blocked with 10% goat serum and 1% bovine serum albumin in PBS-T (PBS with 0.05% Triton X-100) for 2 h at room temperature. Sections were then incubated in Bcl-11B (D6F1) XP rabbit monoclonal antibody (1:100, Cell Signaling) for 2 days in PBS-T (PBS with 0.03% Triton X-100) at 4 °C, followed by incubation with a 555 Alexa Fluor antibody (1:200, ThermoFisher, #A-21428) for 1 h at room temperature.

Sections were then mounted to slides with Vectashield HardSet Antifade Mounting Medium (Vector Laboratories) containing DAPI.

### Image acquisition and analysis

**Fig. 1:**
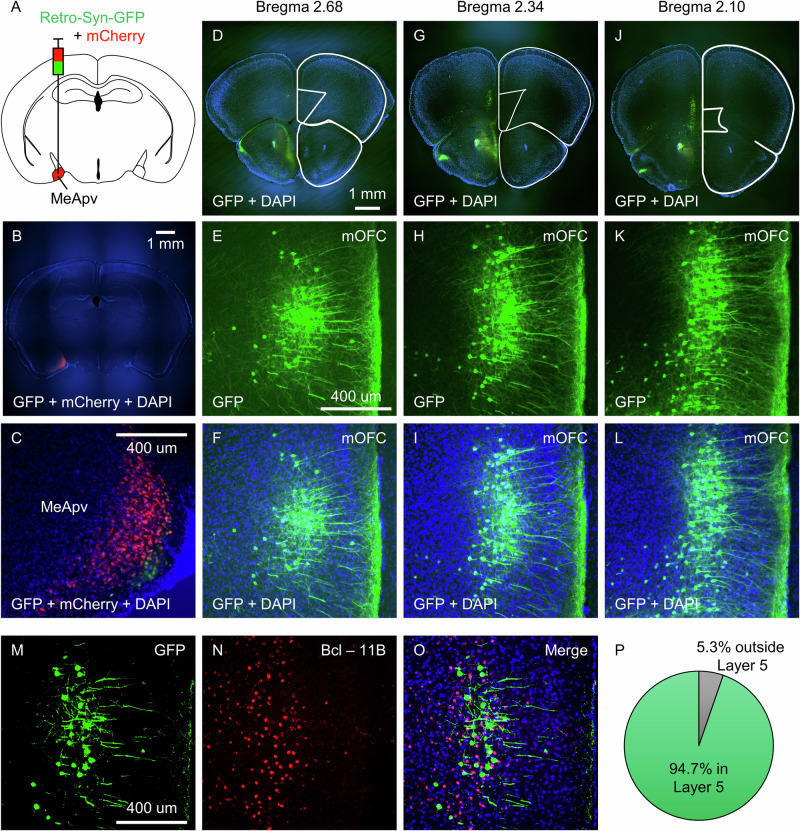
mOFC neurons project onto the MeApv. **A** Injection strategy. Representative low (**B**) and high (**C**)-magnification images of mCherry and retro-hSyn-GFP-expression in the MeApv. Representative low (**D**–**F**) and high (**G**–**L**)-magnification images of retro-hSyn-GFP-expression in the mOFC at various coordinates. **M**–**O** Representative images of the mOFC (Bregma 2.34 mm) expressing retro-hSyn-GFP that were immunolabeled for the Layer 5 marker Bcl-11B. **P** Percentage of GFP-expressing neurons in layer 5 of the mOFC compared to the other layers of the mOFC, as determined by the area labeled by Bcl-11B. All tissue was counterstained with DAPI (blue).

**Fig. 2:**
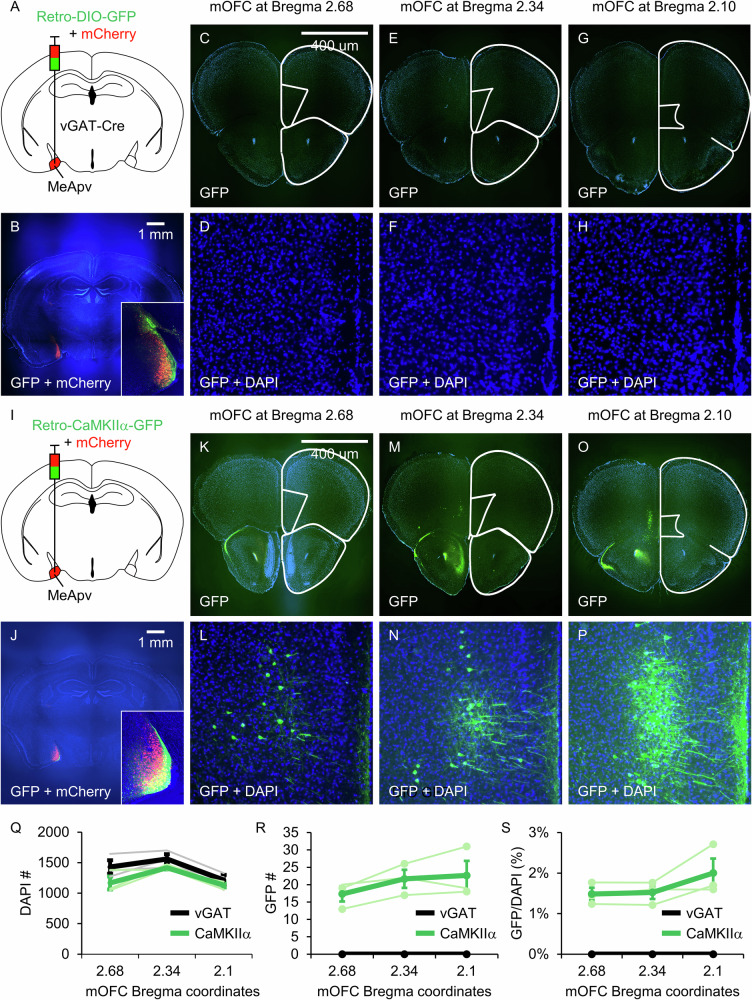
Excitatory, but not inhibitory, mOFC neurons project to the MeApv. **A**, **I** Injection strategy. vGAT-Cre mice were used for *A-H* and WT mice were used for *I-P*. **B**, **J** Representative low- and high-magnification images of the MeApv expressing mCherry and either retro-DIO-GFP-expression in vGAT-Cre mice (**B**) or retro-CaMKIIα-GFP in WT mice. Representative low-magnification images of brain sections containing the mOFC from vGAT-Cre mice injected with retro DIO-GFP (**C**, **E**, **G**) or WT mice injected with retro CaMKIIα-GFP (**K**, **M**, **O**). All tissue was counterstained with DAPI (blue). **C**–**H** and **K**–**P** Representative high-magnification images of the mOFC from vGAT-Cre mice injected with retro DIO-GFP (**D**, **F**, **H**) or WT mice injected with retro CaMKIIα-GFP (**L**, **N**, **P**). All tissue was counterstained with DAPI (blue). **Q**–**S** Quantification of the number of DAPI+ cells (**Q**), GFP+ cells (**R**), and percentage of GFP+ cells in the mOFC at the different coordinates from vGat-Cre mice injected with retro-DIO-GFP (black) or WT mice injected with retro-CaMKIIα-GFP (green) (*n* = 3 mice per condition). Data are mean ± SEM.

Sections were imaged with a fluorescent microscope (EVOS) using a 10x (NA 0.45) objective or a Nikon fluorescent microscope (ECLIPSE Ti2-E) using a 4x (WD 20) objective. To quantify GFP+ cells in the retrograde viral tracing experiments (Figs. 1, 2), GFP+ cells were identified using the “Analyze Particles” function of ImageJ and validated as cells that overlap with DAPI [14, 15, 43–45]. These colocalized GFP + /DAPI cells were automatically counted using the “Multi Measure” function of ImageJ.

To quantify the percentage of GFP+ neurons in cortical Layer 5 of the mOFC, Bcl-11B immunostaining was used [46, 47]. GFP+ cells were classified as either within the Bcl-11B-positive boundary or in the adjacent layers 1-4 and 6 using the ImageJ “Analyze” function.

### Fiber photometry recording

Calcium activity was recorded using a tri-color fiber photometry rig (R820 and R821, RWD Life Science), which houses a 410 nm, 470 nm, and 560 nm LED [43]. The 470 nm and 560 nm LEDs were used to image GCaMP and jRGECO, respectively. The 410 nm LED was used as an isosbestic reference. The three LEDs were routed through a triple band 410/470/560 nm trichroic and 200 μm core, 0.37NA patch cord to a chronically implanted ferrule positioned 0.1 mm above the MeApv or mOFC. Fluorescence returned through the same fiber and was captured by the system’s integrated sCMOS detector at 60 Hz. Behavioral video was acquired simultaneously at 60 fps. The shocks and photometry signals were synchronized using Doric Neuroscience Studio (Doric) by sending transistor–transistor logic (TTL) pulses to both the shock apparatus and the photometry acquisition system, allowing for precise temporal alignment between shock onset and photometry signal acquisition.

Motion artifacts and bleaching were corrected by fitting the 410 nm trace to the 470 nm and 560 nm trace with least squares regression and computing (e.g., ΔF/F = (470 nm – fitted 410 nm)/fitted 410 nm). The resulting trace was z-scored to a 2 s pre-event (foot shock) baseline for each bout. The 2 s post-shock window was chosen because the photometry signal reliably peaked within this interval across trials. Custom written MATLAB scripts were used to generate a ΔF/F raster plot heatmap, z-score photometry trace, and to perform area under the curve (AUC) statistical analyses. Photometry signals and evoked responses were averaged within each animal, and animal means were used for statistical analyses.

### Chemogenetics

To inhibit mOFC^MeApv^ neurons during traumatic stress-induction, mice were delivered intraperitoneal (IP) injections of 2 mg/kg clozapine-n-oxide (CNO, Sigma) or 0.9% saline (Veh) 30 min before foot shock [12]. Mice were then tested for aggression 7 days later (described above).

### Experimental design and statistical analyses

GraphPad Prism software was used for statistical analysis. All analyses were blind to condition. Power analyses for each experiment were calculated using G*power and adjusted based on previous studies [13–15, 43]. All analyses assume a standard deviation of 20%, 1-β of 0.8, and an α of 0.05. Assumptions were checked for all experimental models using the Shapiro–Wilk test of normality and Levene’s test for equality of variance. *P* < 0.05 was considered significant, and all tests were two-tailed. All data were presented as mean ± SEM. Details can be found in Table S2.

## Results

### The MeApv receives input from excitatory, but not inhibitory, mOFC neurons

To examine how traumatic stress activates the MeApv aggression circuit, we began by mapping the inputs to the MeApv using retrograde viral tracing. The MeApv of four-to-seven-week-old mice were injected with retro-AAV encoding GFP under the control of the synapsin promoter (retro-GFP) along with local mCherry (CaMKIIα-mCherry) (Fig. 1A and Fig. S2A). Synapsin restricts transgene expression to neurons [48–50]. The local mCherry virus was used to confirm accurate targeting of the retro-AAV virus to the MeApv (Fig. 1B, C). When we examined the tissue 3-weeks later, we found GFP expression in expected areas such as the accessory olfactory bulb and cortical amygdala [51–53]. but also throughout the mOFC (Fig. 1D–L), a region implicated in emotion regulation and aggression through top-down control of the amygdala [8, 11, 28, 29, 37, 54, 55].

Upon inspection, GFP+ neurons appeared to localize to deeper layers of the mOFC, consistent with cortical output Layer 5 [56, 57]. To confirm that the MeApv-projecting mOFC (mOFC^MeApv^) neurons were predominantly located in Layer 5, we injected retro-AAV-hSyn-GFP into the MeApv and then immunolabeled tissue sections containing the mOFC with the Layer 5 antibody Bcl-11B (DGF1) XP [46, 47]. The GFP+ neurons were quantified within and outside the Bcl-11B defined boundary. Analysis revealed that 94.7% of the mOFC^MeApv^ neurons were found within Layer 5 (Fig. 1M–P).

Historically, authors have speculated that the OFC operates as a “brake” on aggression centers, suggesting the involvement of inhibitory neurons [16–21]. In this conception, traumatic stress could facilitate aggression by somehow inhibiting the mOFC, leading to disinhibition of the MeApv aggression pathway. To assess whether the mOFC^MeApv^ neurons are inhibitory, the MeApv of vGAT-Cre mice was injected with retro-AAV encoding Cre-dependent GFP (retro-DIO-GFP) as well as local mCherry (CaMKIIα-mCherry) (Fig. 2A–B, Fig. S2). vGAT-Cre mice express Cre-recombinase in inhibitory neurons [58]. Thus, allowing us to label just the inhibitory mOFC population with GFP. Interestingly, while we saw GFP expression in various regions, including the MeApv, we saw no GFP+ cells in the mOFC (Fig. 2C–H and 2Q–S), suggesting that the mOFC^MeApv^ neurons are not inhibitory.

To assess whether the mOFC^MeApv^ neurons might be excitatory, we injected the MeApv of wild type mice with retro-AAV encoding GFP under the control of the CaMKIIα promoter (retro-CaMKIIα-GFP) as well as local mCherry (CaMKIIα-mCherry) (Fig. 2I, J and Fig. S2). CaMKIIα restricts transgene expression to excitatory neurons [13, 15, 59]. Broad GFP expression was found throughout the mOFC, indicating that the mOFC^MeApv^ neurons are excitatory (Fig. 2K–P and 2Q–S).

To determine whether excitatory mOFC^MeApv^ neurons are also present in female mice, we performed the same retrograde tracing procedure described above, injecting retro-CaMKIIα-GFP into the MeApv and then examining tissue sections containing the mOFC (Fig. S2 and S3). As with male mice, numerous GFP+ neurons were observed throughout the mOFC (Fig. S3), indicating that female mice possess a similar mOFC–MeApv projection.

These results indicate that excitatory, but not inhibitory, Layer 5 mOFC neurons project to the MeApv, suggesting their involvement in MeApv-related behaviors.

### Foot shock activates the excitatory mOFC^MeApv^ neurons

**Fig. 3:**
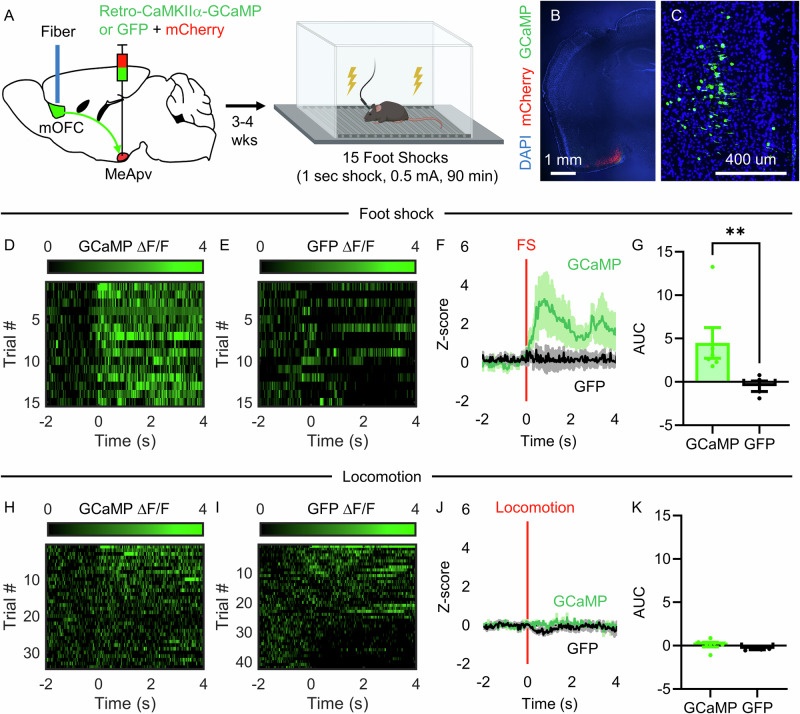
Foot shock activates the excitatory mOFC^MeApv^ neurons. **A** Injection strategy. Mice were unilaterally injected with mCherry and retro-CaMKIIα-GCaMP or GFP control virus into the MeApv and implanted with an optic fiber 0.1 mm above the mOFC. Site-specific recordings were conducted during foot shock three-to-four-weeks later. **B**, **C** Representative images of mCherry and GCaMP-expression in the MeApv and GCaMP-expression in the mOFC (Bregma 2.34 mm). Raster plots showing fluorescence changes in the GCaMP- or GFP-expressing excitatory mOFC^MeApv^ neurons before and after the start of foot shock (**D**, **E**) or non-specific locomotion (**H**, **I**). Average z-scores of fluorescence signal changes in the excitatory mOFC^MeApv^ neurons expressing GCaMP (green trace) or GFP (black trace) before and after the start of foot shock (**F**) or locomotion (**J**) (*n* = 6 and 4). Colored lines indicate group averages and shaded areas indicate SEM. Bar graphs comparing the AUC of foot shock (**G**) or locomotion (**K**) evoked responses (0 to 2 s) from the excitatory mOFC^MeApv^ neurons expressing GCaMP or GFP (*n* = 6 and 4). Data are mean ± SEM. *p < 0.05.

Previous studies from our lab have shown that the excitatory MeApv neurons are strongly activated by traumatic stress (Fig. S1) [12–15, 38, 39]. To assess whether the excitatory mOFC^MeApv^ neurons are similarly activated by traumatic stress, we recorded neural responses to foot shock–our most widely used and effective stressor for inducing aggression–using fiber photometry. Fiber photometry is an in vivo live imaging technique that allows for cell-, pathway-, and site-specific recordings during a temporally aligned stimulus, such as foot shock. To begin, the MeApv of four-to-seven-week-old mice were unilaterally injected with retro-AAV encoding the ultrabright calcium indicator GCaMP8f (retro-CaMKIIα-GCaMP8f) [60]. or GFP control virus (retro-CaMKIIα-GFP), both under the control of the CaMKIIα promoter (Fig. 3A–C and Fig. S2). An optical fiber ferrule was then implanted above the mOFC for site-specific recordings. The photometry recording details can be found in the Materials and Methods section. Only male mice were used for the recordings, as traumatic stress did not increase aggression in female mice in our paradigm (all interaction and main effects and post-hoc analyses were *p* < 0.05, Fig. S4, Table S2).

Analysis of the fiber photometry recordings revealed a clear increase in fluorescence in the excitatory mOFC^MeApv^ neurons immediately following foot shock, indicating robust activation of this population during the stress stimulus (Fig. 3D, 3F). In contrast, fluorescence signals did not change during periods of general locomotion, suggesting that the response was specific to the aversive stimulus rather than movement-related activity. No such stimulus-evoked responses were observed in GFP control animals, which showed stable fluorescence signals during both foot shock and locomotion (Fig. 3E, 3I).

Quantification of these responses using area under the curve (AUC) analysis confirmed that foot shock produced significantly larger calcium responses in GCaMP-expressing mice compared to GFP controls (foot shock, U = 0, *p* = 0.0095. Figure 3G, Table S2). In contrast, locomotion-related activity did not differ between groups (locomotion, t_(6.018)_ = 1.805, *p* = 0.1210, Fig. 3K, Table S2), further indicating that the excitatory mOFC–MeApv neurons are selectively activated by traumatic stress.

Although retrograde tracing in vGAT-Cre mice revealed no GFP+ neurons in the mOFC—suggesting that mOFC^MeApv^ neurons are not inhibitory—we sought to functionally confirm this observation using fiber photometry. vGAT-Cre mice were injected with retro-AAV encoding Cre-dependent GCaMP8f (retro-FLEX-GCaMP8m) or GFP control (retro-DIO-GFP) into the MeApv, and an optical fiber ferrule was implanted above the mOFC (Fig. S5A–C and Fig. S2). In contrast to the robust responses observed in excitatory mOFC–MeApv neurons, no changes in fluorescence were detected in response to foot shock or locomotion in vGAT-Cre mice (Fig. S5D–F and Fig. S5H–J). Consistent with this, AUC analysis revealed no differences between conditions for either foot shock or locomotion (foot shock, t_(3.298)_ = 1.121, *p* = 0.3371, Fig. S5G; locomotion, t_(5.811)_ = 0.6382, *p* = 0.5476, Fig. S5K; Table S2), confirming that the mOFC^MeApv^ neurons are not inhibitory.

These results indicate that foot shock activates excitatory mOFC^MeApv^ neurons, suggestive of their role in traumatic stress-induced aggression.

### Foot shock activates the excitatory mOFC-MeApv pathway

**Fig. 4:**
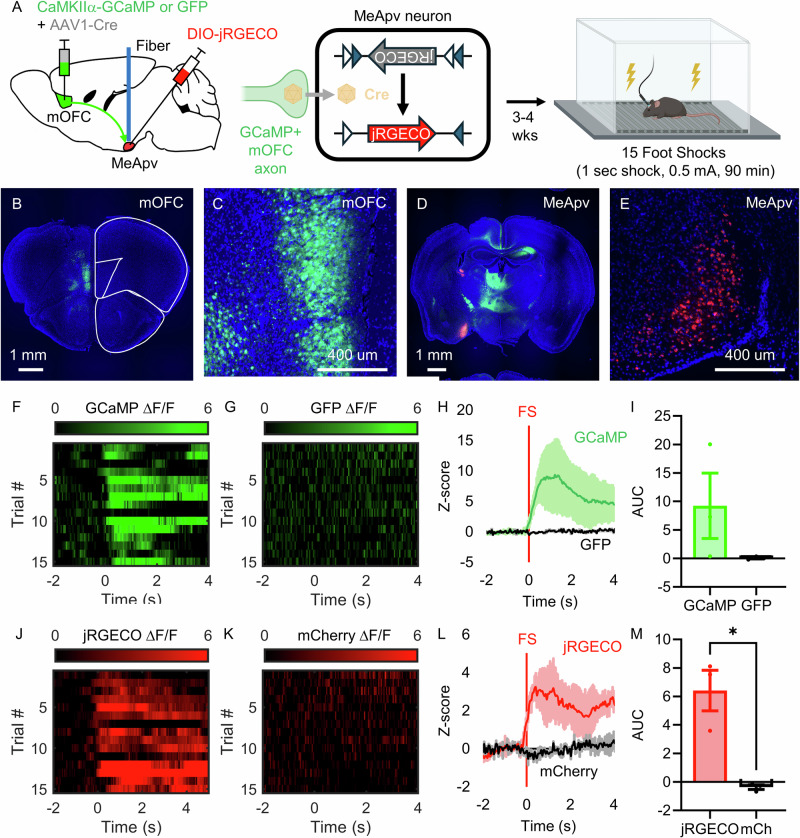
Foot shock activates the excitatory mOFC-MeApv pathway. **A** Injection strategy. Mice were unilaterally injected with AAV1-Cre and CaMKIIα-GCaMP or GFP control virus into the mOFC and DIO-jRGECO or DIO-mCherry control virus into the MeApv, followed by implantation with an optic fiber 0.1 mm above the MeApv. Site-specific recordings were conducted during foot shock 3 to 4 weeks later. Included is a schematic of the anterograde labeling strategy. Representative high- and low-magnification images of GCaMP-expression in the mOFC (**B**, **C** Bregma 2.34 mm) and jRGECO-expression in the MeApv (**D**, **E**). Raster plots showing fluorescence changes in the excitatory mOFC^MeApv^ axons expressing GCaMP or GFP control virus (**F**, **G**) and the mOFC-receiving MeApv neurons expressing jRGECO or mCherry control virus (**J**, **K**) before and after the start of foot shock. Average z-scores of fluorescence signal changes in the excitatory mOFC^MeApv^ axons expressing GCaMP (green) or GFP control (black) (**H**) and mOFC-receiving MeApv neurons expressing jRGECO (red) or mCherry control (black) (**L**) before and after the start of foot shock (*n* = 3 mice per condition). Colored lines indicate group averages and shaded areas indicate SEM. Bar graphs comparing the AUC of foot shock-evoked responses (0 to 2 s) from the excitatory mOFC^MeApv^ axons expressing GCaMP or GFP control (**I**) and mOFC-receiving MeApv neurons expressing jRGECO or mCherry control (**M**) (*n* = 3 mice per condition). Data are mean ± SEM. *p < 0.05.

We next wanted to confirm that the mOFC forms synaptic connections with the MeApv and that both are activated by traumatic stress. To do this, we used anterograde viral tracing and dual-color fiber photometry. Mice were injected with (i) AAV1-Cre–which has anterograde properties–and CaMKIIα-GCaMP6f or CaMKIIα-GFP control into the mOFC, followed by (ii) Cre-dependent jRGECO1a (FLEX-jRGECO) or Cre-dependent mCherry control (DIO-mCherry) into the MeApv (Fig. 4A and Fig. S2). jRGECO1a is an ultrafast, red-shifted calcium sensor ideal for recording neural responses to foot shock [61]. We then implanted an optical fiber ferrule above the MeApv to simultaneously record foot shock-evoked responses from the excitatory mOFC^MeApv^ axons and mOFC-receiving MeApv neurons.

It should be noted that while AAV1 is commonly used for anterograde transsynaptic labeling, it can exhibit limited retrograde transport [62]. and thus some of the jRGECO signal may reflect mOFC-projecting MeApv neurons. In addition, while we observed some viral expression outside the mOFC, including in the olfactory bulb, the majority of expression was localized to the mOFC (Fig. 4B, C). Notably, we did not observe GFP fluorescence in the posterior dorsal MeA (MeApd), a region implicated in real-time attack behavior [63].

As expected, we observed robust jRGECO expression in the MeApv (Fig. 4D, E). During foot shock, both the excitatory mOFC^MeApv^ axons (GCaMP signal, Fig. 4F, 4G) and the mOFC-receiving MeApv neurons (jRGECO signal, Fig. 4J, 4L) exhibited rapid increases in fluorescence aligned to shock onset, indicating activation of both the presynaptic mOFC inputs and their downstream MeApv targets. In contrast, no such changes were observed during general locomotion (Figure. S6) or in GFP/mCherry-expressing control animals (Fig. 4G–H, 4K–L).

AUC analysis revealed a significant increase in foot shock-evoked activity in jRGECO-expressing MeApv neurons compared to mCherry controls (*t* = 3.680, *p* = 0.0348, Fig. 4M; Table S2), and a non-significant difference between GCaMP-expressing mOFC axons compared to GFP controls (t_(2.003)_ = 1.593, *p* = 0.2520, Fig. 4I; Table S2). No differences were observed between groups during locomotion for either signal (GCaMP vs GFP, t_(2.624)_ = 0.026, *p* = 0.9811, Fig. S6D; jRGECO vs mCherry, t_(3.685)_ = 0.8002, *p* = 0.4720, Fig. S6H; Table S2).

Together, these findings demonstrate that traumatic stress engages both the excitatory mOFC axons and their downstream MeApv targets, consistent with activation of the excitatory mOFC–MeApv pathway.

### Excitatory mOFC^MeApv^ neurons mediate traumatic stress-induced aggression

**Fig. 5:**
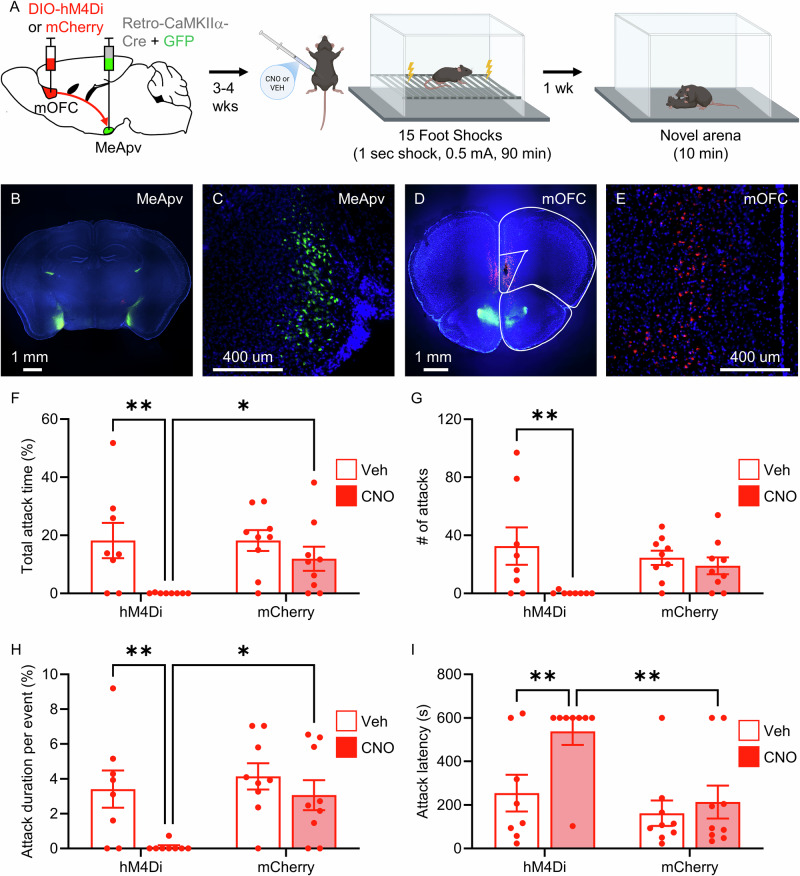
Traumatic stress-induced aggression depends on the activation of the excitatory mOFC^MeApv^ neurons. **A** Experimental schedule. Mice were bilaterally injected with GFP and Retro-CaMKIIα-Cre into the MeApv and DIO-hM4Di or mCherry control virus into the mOFC, followed three-to-four weeks later by IP injections of 2 mg/kg CNO 30 min before receiving the foot shocks. A subset of controls were injected with CaMKIIα-mCherry into the mOFC alone. Aggression was tested 1 week later. Representative high- and low- magnification images of GFP-expression in the MeApv (**B**, **C**) and hM4Di-expression in the mOFC (**D**, **E** Bregma 2.34 mm). **F**–**I** Quantification of aggressive behavior from mice expressing hM4Di or mCherry control virus and treated with vehicle (Veh) or CNO 30 min before receiving foot shocks. Cre-dependent and CaMKIIα-mCherry controls produced comparable levels of aggression for all metrics (*p* > 0.05), and so were pooled (*n* = 8, 8, 9, 9). Mean +/− SEM. *p < 0.05, **p < 0.01.

To determine the causal role of the excitatory mOFC^MeApv^ neurons in traumatic stress-induced aggression, we used a chemogenetic approach, which allows for cell-specific control of neural activity using a virally-transduced drug receptor [64]. To begin, mice were injected with retro-AAV-CaMKIIα 0⊡4-Cre and CaMKIIα-GFP into the MeApv followed by AAV9 encoding Cre-dependent hM4Di (DIO-hM4Di) into the mOFC, or CaMKIIα-mCherry into the mOFC alone (Fig. 5A and Fig. S2). hM4Di is an inhibitory Designer Receptors Exclusively Activated by Designer Drugs (DREADD) receptor used to chemogenetically control neurons in vivo. The mCherry viruses were used as DREADD receptor controls. As we and others have shown, hM4Di inhibits neurons when bound by its synthetic ligand clozapine-N-oxide (CNO) [12, 14, 43, 64]. The combination of the above viruses allowed us to selectively express hM4Di in the excitatory mOFC^MeApv^ neurons for control during foot shock (Fig. 5B–E).

Three-weeks later, experimental mice received IP injections of 2 mg/kg CNO or vehicle 30 min before foot shock. When we tested them for aggression in a novel cage 7 days later, we found that chemogenetic inhibition produced a marked reduction in aggressive behavior (Fig. 5F–I). While two-way ANOVAs revealed significant main effects of CNO treatment across all aggression measures (Attack time, F(1,30) = 8.949, *p* = 0.0055, Fig. 5F; Attack number, F(1,30) = 6.729, *p* = 0.0145, Fig. 5G; Duration per attack, F(1,30) = 7.725, *p* = 0.0093, Fig. 5H; Attack latency, F(1,30) = 5.604, *p* = 0.0246, Fig. 5I; Table S2) and significant main effects of viral condition for two metrics (Attack time, F(1,30) = 2.125, *p* = 0.1553, Fig. 5F; Attack number, F(1,30) = 0.5246, *p* = 0.4745, Fig. 5G; Duration per attack, F(1,30) = 5.487, *p* = 0.0260, Fig. 5H; Attack latency, F(1,30) = 8.685, *p* = 0.0062, Fig. 5I; Table S2), no interaction effects reached significance (Table S2). Nevertheless, post hoc analysis indicated that CNO-treatment nearly abolished all aggression in the hM4Di-expressing mice, whereas vehicle-treated and mCherry controls continued to display robust aggression, commensurate with our previous studies and confirmed here (Fig. S4; Table S2) [13, 15, 39].

We have repeatedly found that while the excitatory MeApv neurons control traumatic stress-induced aggression, they have no effect on non-aggressive social interaction [13–15, 38, 39]. To test whether the excitatory mOFC^MeApv^ neurons similarly spare social behavior after traumatic stress, we also measured the amount of non-aggressive social interaction in our aggression experiments. As with our MeApv manipulations, chemogenetically inhibiting the excitatory mOFC^MeApv^ neurons had no effect on total interaction time, number of social interactions, or average duration of a social interaction (Fig. S7A–C, Table S2). However, we did observe an increase in the latency to the first social interaction (Fig. S7D: Interaction effect, F(1,30) = 6.541, *p* = 0.0158; Treatment main effect, F(1,30) = 7.853, *p* = 0.0088; Viral main effect, F(1,30) = 3.814, *p* = 0.0602; Table S2), suggesting that the excitatory mOFC^MeApv^ neurons control some level of non-aggressive social behavior.

To determine the role of excitatory mOFC neurons more broadly in traumatic stress-induced behavior, we injected CaMKIIα-hM4Di or CaMKIIα-mCherry control virus directly into the mOFC and three weeks later administered IP injections of CNO 30 min before foot shock (Fig. S8A and Fig. S2). Aggression was tested 7 days later. Consistent with our projection-specific manipulations, chemogenetic inhibition of excitatory mOFC neurons significantly reduced aggression in hM4Di-expressing mice compared to mCherry controls (Attack time, U = 0, p = 0.0003, Fig. S8B; Attack number, U = 1, *p* = 0.0006, Fig. S8C; Duration per attack, U = 0, *p* = 0.0003, Fig. S8D; Attack latency, U = 5, *p *= 0.0057, Fig. S8E; Table S2).

However, in contrast to the selective effects observed with mOFC^MeApv^ inhibition, global inhibition of excitatory mOFC neurons increased total interaction time and the average duration of social interactions (Interaction bout time, U = 10, *p* = 0.0401, Fig. S8F; Interaction bout number, t_(11.90)_ = 1.810, *p* = 0.0956, Fig. S8G; Duration per interaction bout, t_(9.047)_ = 2.417, *p* = 0.0386, Fig. S8H; Interaction bout latency, U = 27, *p* = 0.9259, Fig. S8I; Table S2). These findings suggest that excitatory mOFC neurons broadly regulate social behavior, whereas the mOFC^MeApv^ neurons more selectively mediate traumatic stress-induced aggression.

In sum, these findings demonstrate that traumatic stress recruits excitatory mOFC^MeApv^ neurons to drive long-lasting aggression.

## Discussion

### Summary of results

This study identifies a cortical–amygdala circuit that contributes to traumatic stress–induced aggression. Using viral tracing, we show that excitatory—but not inhibitory—Layer 5 neurons in the mOFC project to the MeApv. Fiber photometry revealed that traumatic stress activates these mOFC^MeApv^ neurons and their downstream MeApv targets, while chemogenetic inhibition of this pathway during stress attenuated the subsequent increase in aggression without affecting non-aggressive social interaction, consistent with prior work showing that the MeApv–VmHvl pathway drives stress-induced aggression [13–15].

In contrast, global inhibition of excitatory mOFC neurons suppressed aggressive behavior but surprisingly increased some non-aggressive social behaviors, suggesting that while the mOFC broadly regulates social behavior under stress, the aggression phenotype arises specifically through its projections to the MeApv. Together, these findings demonstrate that activation of an excitatory mOFC^MeApv^ pathway during traumatic stress is necessary for the emergence of long-lasting aggression and supports a reinterpretation of OFC function from a simple inhibitory “brake” on aggression to a circuit-specific regulator whose dysregulation can promote pathological aggression.

### Revising the function of the OFC in aggression regulation

Our findings challenge the classical view that the OFC primarily inhibits subcortical aggression centers—a framework rooted in early lesion and neuropsychological studies, including the case of Phineas Gage and subsequent reports of disinhibition following frontal damage [16–21]. Human neuroimaging studies similarly associate reduced OFC activity or structural deficits with impulsivity and aggression [11, 22–29]. while stress-induced OFC disruption predicts increased aggression and abnormal OFC–amygdala connectivity [65–69]. Together, these findings have supported a model in which the OFC acts as a cortical brake on limbic-driven behavior.

However, such interpretations reflect the limits of early lesion and pharmacological approaches, which broadly disrupt local tissue, inputs, and fibers of passage. For example, aspiration lesions in macaques impair reversal learning, but fiber-sparing excitotoxic lesions do not [17, 32]. Similarly, muscimol inactivation of the OFC prior to behavioral testing increased post-shock fighting [23]. But these manipulations do not distinguish projection-defined populations or the timing of OFC engagement. Similarly, human lesion and imaging studies lack the resolution to determine whether OFC–amygdala interactions are inhibitory, excitatory, or context-dependent [17, 70, 71]. Thus, while these studies established an important role for the OFC in aggression control, they do not resolve how specific OFC circuits regulate downstream targets.

Our data provide this resolution, showing that a defined population of excitatory mOFC^MeApv^ neurons is activated during traumatic stress and is required for the subsequent increase in aggression. Rather than supporting a strict inhibitory “brake” model, these findings suggest that the mOFC regulates amygdala activity in a context-dependent manner, with traumatic stress biasing this regulation toward maladaptive outcomes. In this framework, the mOFC does not directly drive aggression, but instead modulates the state of downstream circuits in a way that can promote or constrain aggressive behavior depending on prior experience. Consistent with this, neuroimaging studies show that early trauma reverses the typical negative correlation between OFC and amygdala volume, suggesting maladaptive coupling after stress [68].

Mechanistically, our data support a model in which traumatic stress hyperactivates the mOFC-MeApv pathway, thereby potentiating the MeApv-VmHvl synapses previously shown to mediate experience-dependent aggression (Fig. S9). This provides a potential explanation for how stress can “prime” subcortical circuits, biasing behavioral output toward excessive aggression during a subsequent social challenge rather than directly triggering attack behavior. Consistent with this interpretation, mOFC projections selectively target the MeApv and not the MeApd, suggesting preferential engagement of circuits involved in experience-dependent aggression escalation rather than those controlling real-time attack execution [15, 63]. Together, these findings support a model in which traumatic stress hijacks normal mOFC regulation of amygdala circuits, leading to persistent changes in circuit function that bias behavior toward maladaptive aggression.

### Limitations

Several limitations should be considered. First, while we elected to use foot shock as a reproducible model of traumatic stress, it is less ethologically relevant than acute social defeat, a stressor we show also produces long-lasting aggression through activation of the MeApv-VmHvl pathway [14]. However, while social defeat is conceptually closer to real-world trauma than foot shock, it introduces high variability in aggression escalation and MeApv-VmHvl pathway activation due to inconsistent attack severity and duration [15, 72]. making foot shock a preferable stress-induction strategy for the present study. That said, acute social defeat would establish the generality of the mOFC-MeApv pathway across stress-induced aggression paradigms.

Second, the behavioral effect was sex-specific, as female mice did not exhibit increased aggression following stress–consistent with previous reports [73]. despite possessing similar mOFC-MeApv circuitry. These findings suggest that traumatic stress may differentially recruit or modify the OFC–MeApv pathway across sexes, potentially due to hormonal or molecular factors. Consistent with this idea, prior studies have shown that MeA circuits exhibit strong sexual dimorphism in both connectivity and neuromodulatory control [74, 75]. Future work using more aggressive female strains [76]. or manipulating hormonal state could help determine whether differences in circuit recruitment underlie the sex-specific behavioral phenotype.

Third, while we focused on the mOFC, our retrograde mapping also identified projections from the lateral OFC and insular cortex—regions strongly implicated in emotional appraisal, impulse control, and social cognition [17, 77–82]. Future work could examine whether these regions converge on or modulate the MeApv in similar ways.

Fourth, our viral approaches have inherent limitations. Retrograde AAVs may not label all projecting neurons, and thus, we cannot fully exclude the presence of sparse inhibitory projections. Similarly, while AAV1 is commonly used for anterograde tracing, it can exhibit limited retrograde transport [62]. raising the possibility that a subset of labeled MeApv neurons reflects mOFC-projecting cells rather than exclusively mOFC-MeApv connectivity. Our dual-virus photometry experiments require larger injection volumes and extended expression time, resulting in some spread beyond the mOFC. While these factors could introduce off-target labeling, multiple lines of evidence including projection-specific recordings, terminal activity in the MeApv, and chemogenetic inhibition of mOFC-MeApv neurons support a primary role for this pathway in stress-induced aggression.

Fifth, the Layer 5 excitatory mOFC projection neurons likely collateralize to multiple downstream targets. Although our retrograde strategies define MeApv-projecting neurons, they do not exclude the possibility that these neurons influence behavior through additional projections. Future studies using terminal-restricted or intersectional approaches will be required to isolate pathway-specific contributions.

Sixth, while our data establish necessity, sufficiency remains to be tested. Future studies could assess whether activating the excitatory mOFC^MeApv^ neurons during traumatic stress enhances aggression in males while promoting aggression in females, further demonstrating the critical role of the mOFC-MeApv pathway in maladaptive attack behavior.

Finally, while we show that the mOFC-MeApv pathway is activated during traumatic stress and is necessary for the subsequent increase in aggression, we did not assess its activity during the aggression test itself. It remains possible that traumatic stress alters the function of this pathway during a socially challenging situation to bias aggression responding. Future studies should address this.

### Clinical Implications

Beyond its mechanistic contribution, these findings have important translational implications, offering exciting therapeutic possibilities. The OFC’s superficial and accessible location makes it a strong candidate for transcranial magnetic stimulation (TMS), which can modulate OFC activity through the lateral PFC, a current TMS target to treat depression, anxiety, and PTSD [66, 83–85]. Targeted stimulation could potentially restore balance in the OFC–amygdala pathway, mitigating aggression by recalibrating top-down control over limbic networks. Such an approach would represent a non-invasive avenue for treating stress-related aggression—a condition for which few effective therapies exist.

In parallel, our prior work shows that NMDA receptor antagonists reliably blunt the stress-induced synaptic- and structural plasticity changes in the MeApv–VmHvl pathway that bias long-lasting aggression in mice [12, 15, 39, 86, 87]. Moreover, NMDA receptor blockade within the OFC can reverse stress-driven behavioral dysfunction [88]. raising the possibility that targeting glutamatergic signaling in the mOFC^MeApv^ pathway could also be leveraged to treat traumatic stress-induced aggression. Future studies should directly test this therapeutic avenue.

## Summary

This study demonstrates that traumatic stress recruits an excitatory mOFC^MeApv^ circuit to drive long-lasting aggression. By combining viral tracing, in vivo calcium imaging, and chemogenetic inhibition, we establish a causal role for this cortical–amygdala pathway in stress-induced behavioral change. These findings challenge the historical “inhibitory OFC” model, reframing the OFC as a context-dependent modulator whose dysregulation amplifies emotional drives. Ultimately, our work identifies a precise cortical node linking trauma to pathological aggression and suggests that restoring OFC function could be a viable therapeutic strategy.

## Supplementary information

Supplementary Statistics Table

Supplementary Material

## Data Availability

The datasets generated and analyzed during the current study are available from the corresponding author upon request. Source data underlying all figures, including behavioral metrics, fiber photometry recordings, and statistical analyses, are provided in the Supplementary Information (Supplementary Table 1). Detailed information on viral constructs used in the study is also available in Supplementary Table 2. Custom MATLAB scripts used for photometry analyses are available from the corresponding author upon request.
