# Supplementary material for: Traumatic stress recruits an excitatory orbitofrontal–amygdala pathway to drive maladaptive aggression
Source: Neuropsychopharmacology. 2026 May 6;51(9):1610–22. doi: 10.1038/s41386-026-02432-z (PMC13388918; doi:10.1038/s41386-026-02432-z)
Supplement: Supplementary file 2 — Supplementary Material [file 41386_2026_2432_MOESM2_ESM.pdf]

**Supplementary Table 1. Supplementary statistics table for all data in the manuscript.** See separate excel table.

**Supplementary Table 2. Viruses used in the manuscript.**

| Virus | Abbreviation | Addgene Product # | Titer (vg/ml) |
|---|---|---|---|
| retro-pAAV-hSyn-EGFP | retro-GFP | 50465-AAVrg | $1.9 \times 10^{13}$ |
| retro-pAAV-hSyn-DIO-EGFP | retro-DIO-GFP | 50457-AAVrg | $1.3 \times 10^{13}$ |
| retro-pAAV-CaMKIIα-EGFP | retro-CaMKIIα-GFP | 50469-AAVrg | $2.0 \times 10^{13}$ |
| pENN-AAV1-hSyn-Cre-WPRE-hGH | AAV1-Cre | 105553-AAV1 | $2.1 \times 10^{13}$ |
| pAAV9-CaMKIIα-EGFP | CaMKIIα-GFP | 50469-AAV9 | $2.3 \times10^{13}$ |
| pAAV9-hSyn-DIO-mcherry | DIO-mCherry | 50459-AAV9 | $2.3 \times 10^{13}$ |
| pAAV9-CaMKIIα-mCherry | CaMKIIa-mCherry | 114469-AAV9 | $1.8 \times 10^{13}$ |
| retro-pGP-AAV-Syn-FLEX-jGCaMP8m-WPRE | retro-FLEX-GCaMP8m | 162378-AAVrg | $2.0 \times 10^{13}$ |
| retro-pAAV-CaMKIIα-jGCaMP8f-WPRE | retro-CaMKIIα-GCaMP8f | 176750-AAVrg | $2.1 \times 10^{13}$ |
| AAV9-CaMKIIα-GCaMP6f-WPRE-SV40 | CaMKIIα-GCaMP6f | 100834-AAV9 | $2.3x10^{13}$ |
| pAAV1-syn-Flex-NES-jRGECO1a-WPRE-SV40 | FLEX-jRGECO | 100853-AAVrg | $2.3 \times 10^{13}$ |
| pAAV9-hSyn-DIO-mCherry | DIO-mCherry | 50459-AAV9 | $2.3 \times 10^{13}$ |
| retro-pENN-AAV-CaMKII 0.4-Cre-SV40 | retro-AAV-Cre | 105558-AAVrg | $2.0 \times 10^{13}$ |
| pAAV9-CaMKIIα-hM4D(Gi)-mCherry | hM4Di | 50477-AAV9 | $9.9x10^{12}$ |
| pAAV9-hSyn-EGFP | hSyn-GFP | 50465-AAV9 | 1.9x10^13 |
| pAAV9-hSyn-DIO-hM4D(Gi)-mCherry | DIO-hM4Di | 50475-AAV9 | $1.6 \times 10^{13}$ |

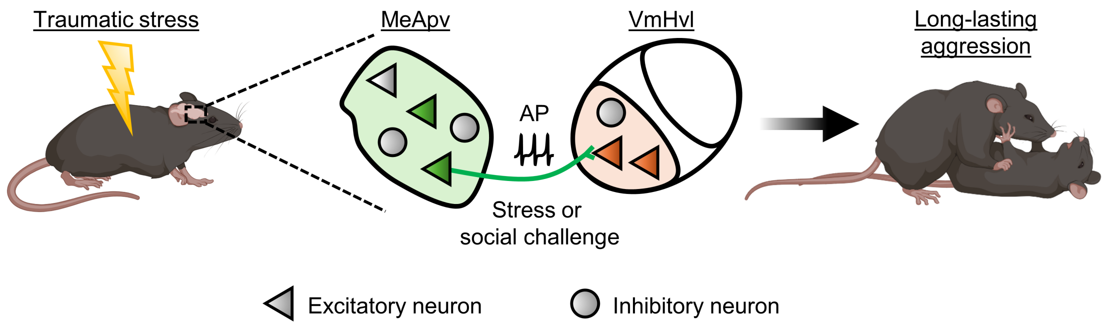


**Supplementary Fig. 1. Summary of previous work.** Traumatic stress drives aggression by activating and potentiating an excitatory MeApv–VmHvl pathway.

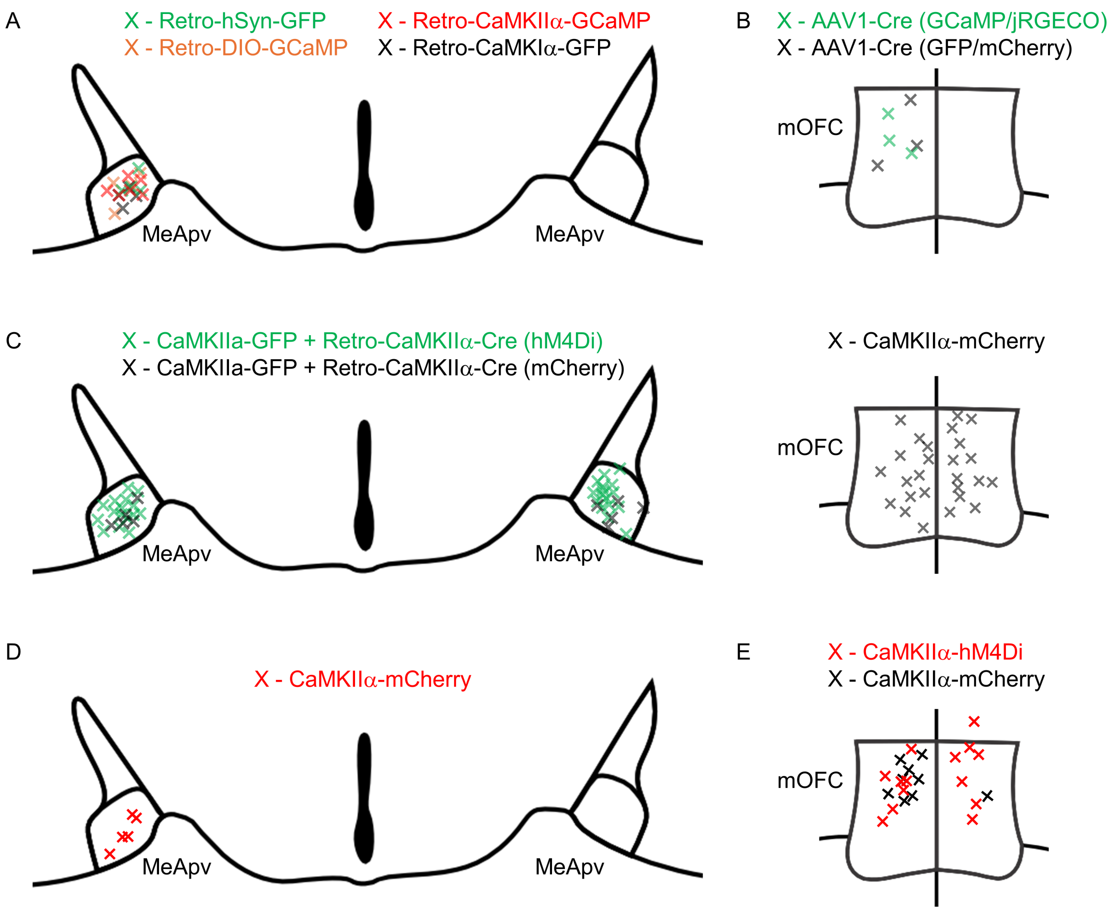


**Supplementary Fig. 2. Atlas images depicting viral targeting.** (A) Injections for Fig. 1-3, S3 and S4. (B) Injections for Fig. 4 (B), and Fig. 5 and S6. (C) Injections for Fig. 5 and S7.

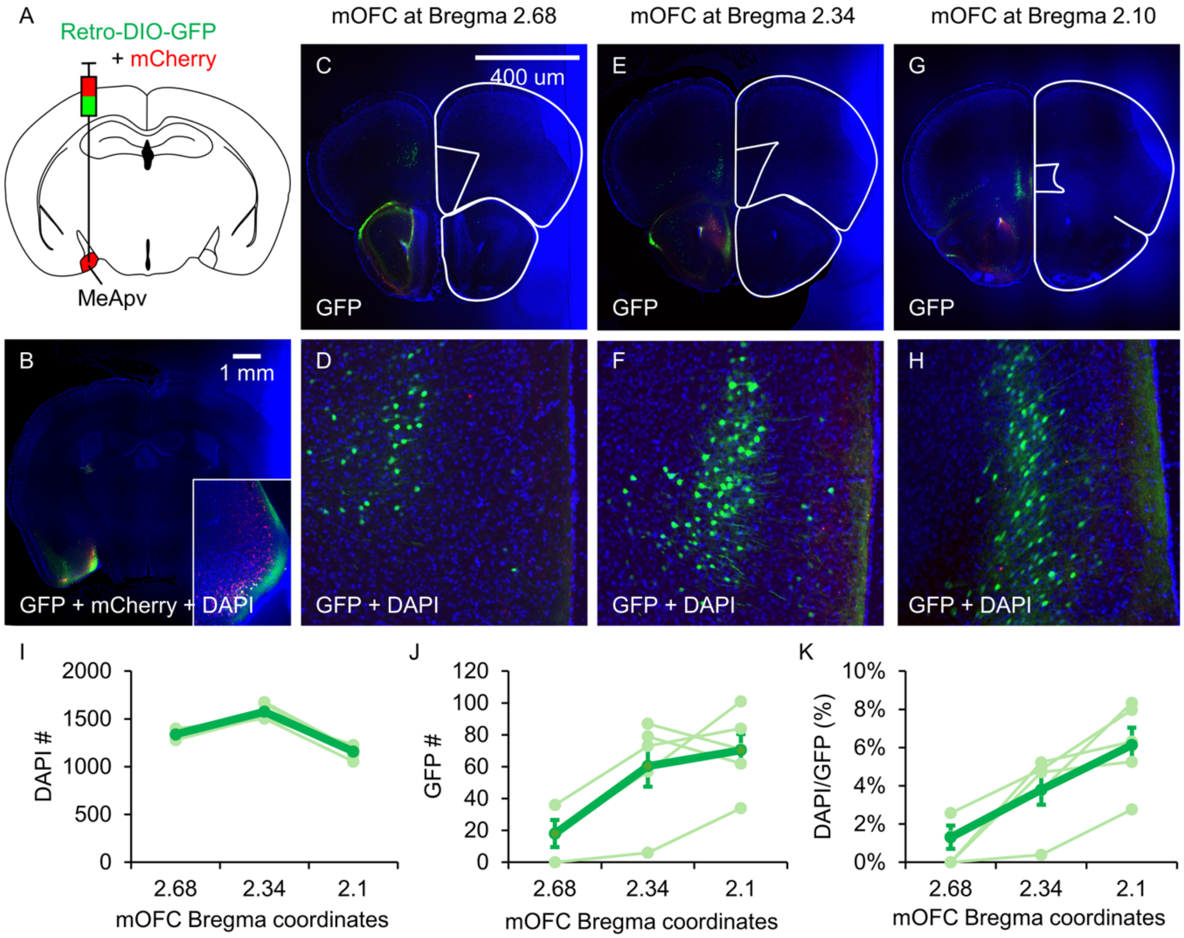


**Supplementary Fig. 3. Excitatory mOFC neurons project to the MeApv of female mice.** (A-B) Injection strategy and representative image of the MeApv of WT mice expressing mCherry and retro-CaMKIIα-GFP. (C-H) Representative low (C, E, G) and high (D, F, H) magnification images of brain sections containing the mOFC. All tissue was counterstained with DAPI (blue). (I-K) Quantification of the number of DAPI+ cells (I), GFP+ cells (J), and percentage of GFP+ cells (K) in the mOFC at the different coordinates (n = 5 mice). Mean +/- SEM.

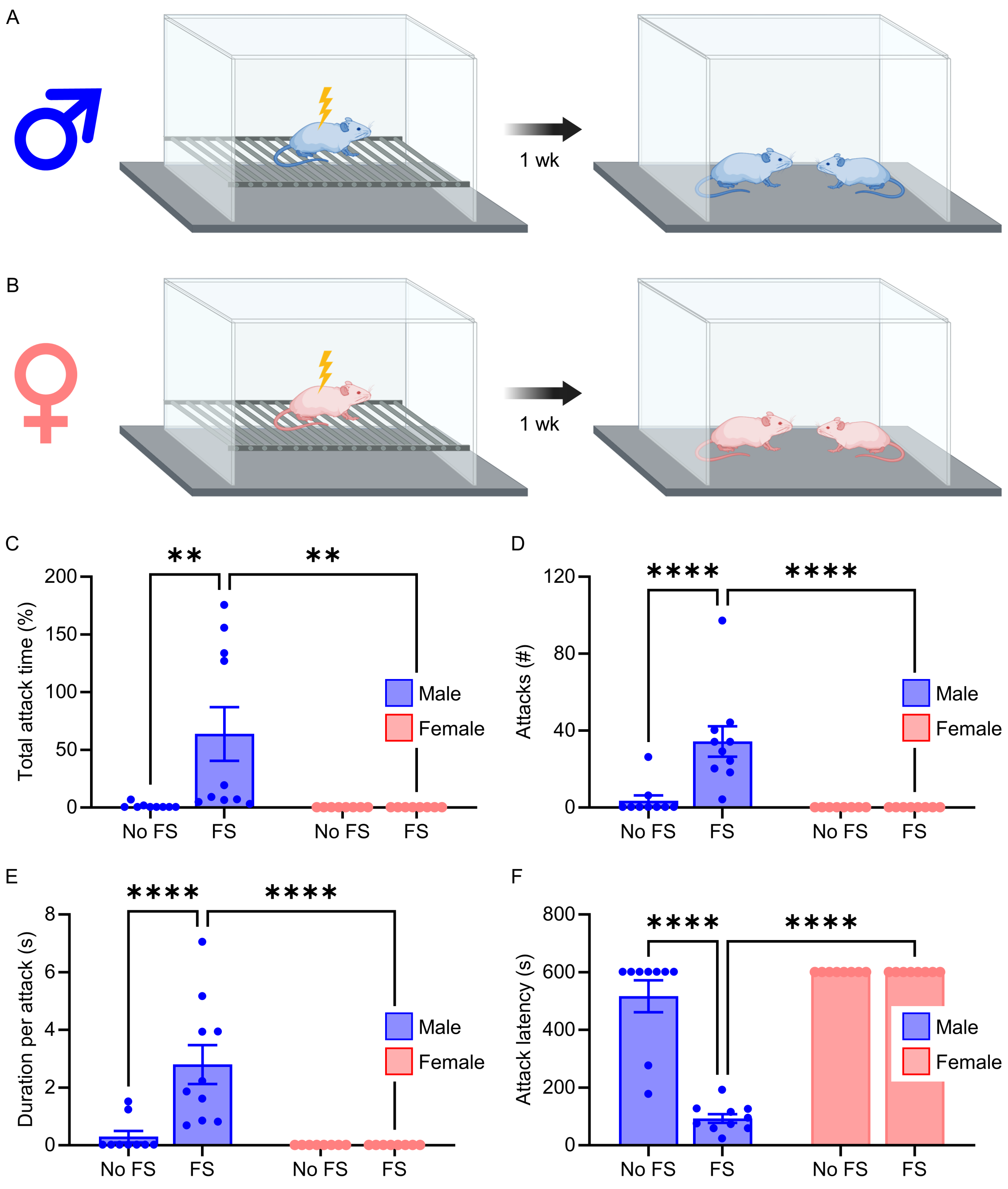


**Supplementary Fig. 4. Traumatic stress promotes long-lasting aggression in males, but not females.** (A-B) Paradigm for testing the impact of traumatic stress on long-lasting aggression in males (A) and females (B). (C-F) Quantification of aggressive behavior from male and female mice exposed to foot shock or control (placed in the foot shock box without receiving foot shocks) (n = 9, 10, 8, 8). Mean +/- SEM. **p<0.01, ****p<0.0001.

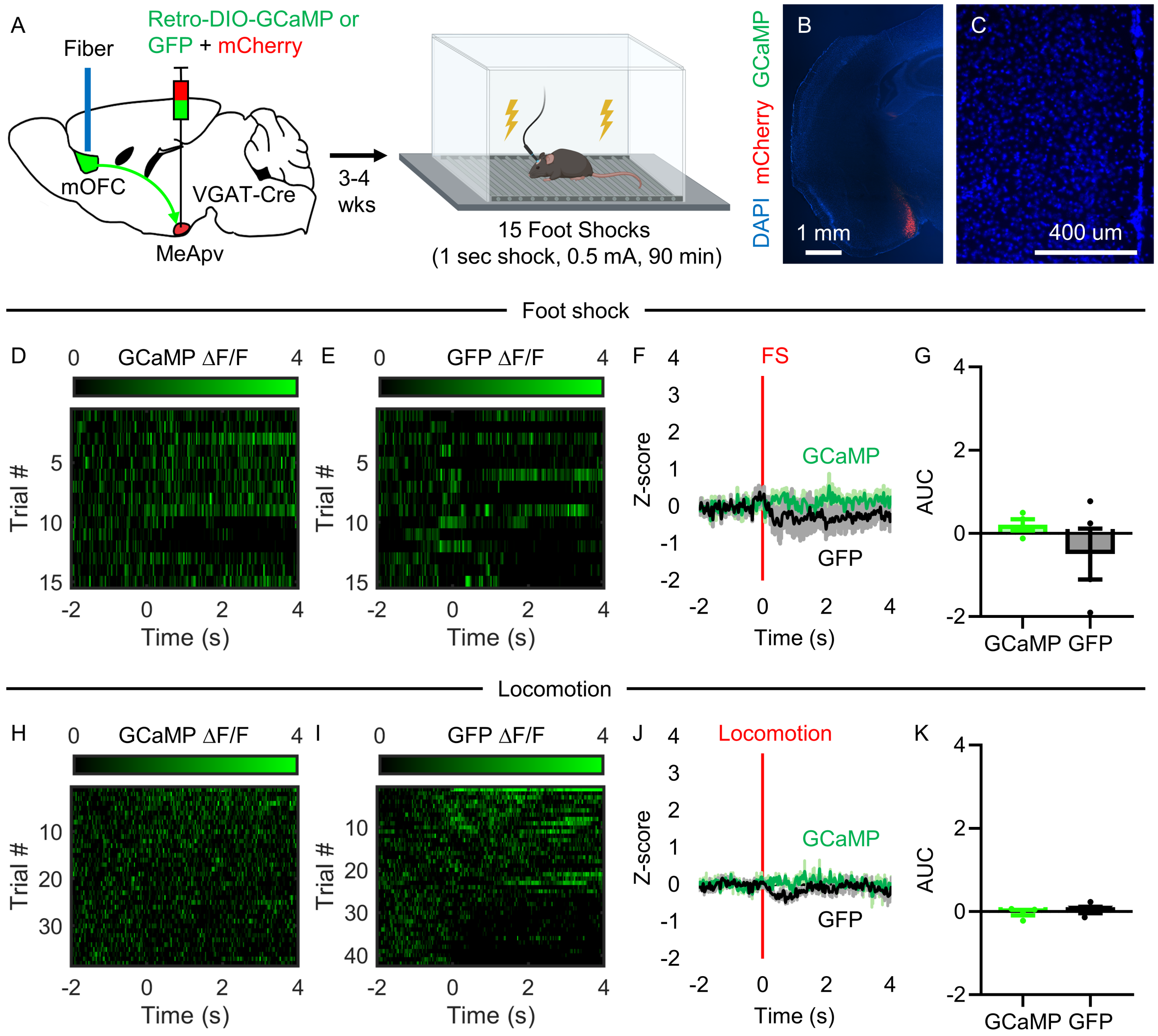


**Supplementary Fig. 5. Confirmation that the mOFC has no inhibitory neurons that project to the MeApv.** (A) Injection strategy. vGAT-Cre mice were unilaterally injected with mCherry and retro-DIO-GCaMP or GFP control virus into the MeApv and implanted with an optic fiber 0.1 mm above the mOFC. Site-specific recordings were conducted during foot shock three-to-four-weeks later. (B-C) Representative images showing mCherry-expression in the MeApv but no GCaMP-expression in the mOFC (Bregma 2.34 mm). (D-E and H-I) Raster plots showing fluorescence changes in the mOFC before and after the start of foot shock (D-E) or non-specific locomotion (H-I). (F and J)

Average z-score of fluorescence signal changes in the excitatory $mOFC^{MeApv}$ neurons expressing DIO-GCaMP (green trace) or DIO-GFP control (black trace) before and after the start of foot shock (F) or locomotion (J) (n = 4 mice per condition). Colored lines indicate group averages and shaded areas indicate SEM. (G and K) Bar graphs comparing the AUC of foot shock (G) or locomotion (K) evoked responses (0 to 2 s) from vGAT-Cre mice injected with DIO-GCaMP or DIO-GFP control (n = 4 mice per condition). Data are mean ± SEM.

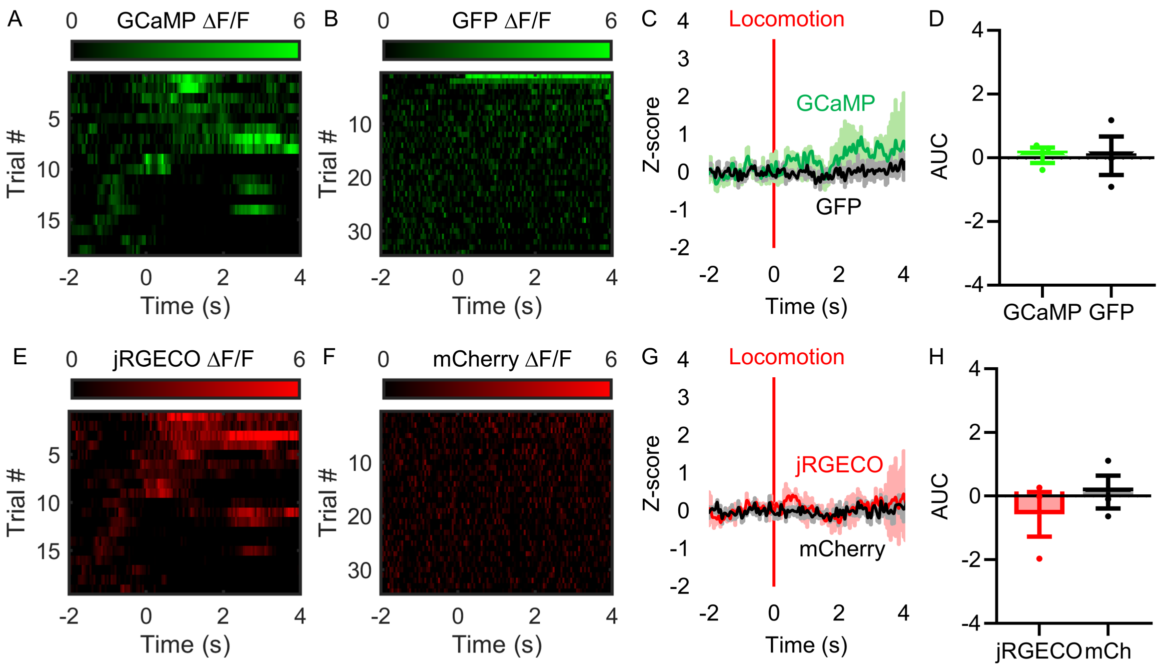


**Supplementary Fig. 6. Locomotion-evoked calcium response from the excitatory mOFC-MeApv pathway, related to Fig. 4.** (A-B and E-F) Raster plots showing fluorescence changes in the excitatory $mOFC^{MeApv}$ axons expressing GCaMP or GFP control (A-B) and mOFC-receiving MeApv neurons expressing jRGECO or mCherry control (E-G) before and after the start of non-specific locomotion. (C and G) Average z-scores of fluorescence signal changes in the excitatory $mOFC^{MeApv}$ axons expressing GCaMP (green) or GFP control (black) (C) and mOFC-receiving MeApv neurons expressing jRGECO (red) or mCherry control (black) (G) before and after the start of non-specific locomotion (n = 3 mice per condition). Colored lines indicate group averages and shaded areas indicate SEM. (D and H) Bar graphs comparing the AUC of non-specific locomotion-evoked responses (0 to 2 s) from the excitatory $mOFC^{MeApv}$ axons expressing GCaMP or GFP control (D) and mOFC-receiving MeApv neurons expressing jRGECO or mCherry control (H) (n = 3 mice per condition). Data are mean ± SEM.

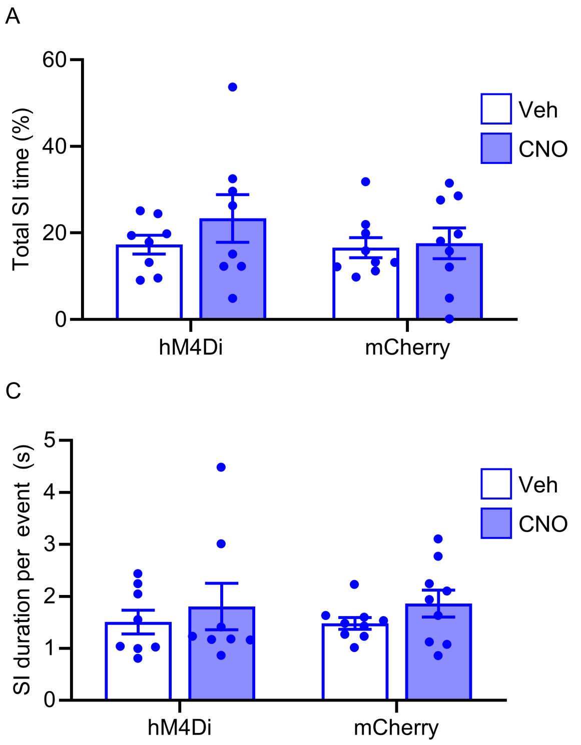


**Supplementary Fig. 7. Inhibiting the excitatory mOFC$^{MeApv}$ neurons during traumatic stress does not affect non-aggressive social interaction (SI).** (A-D) Quantification of non-aggressive social behavior from mice expressing hM4Di or mCherry control virus and treated with vehicle (Veh) or CNO 30 min before the receiving foot shocks (n = 8, 8, 9, 9). Mean +/- SEM. **p<0.01, ***p<0.001.

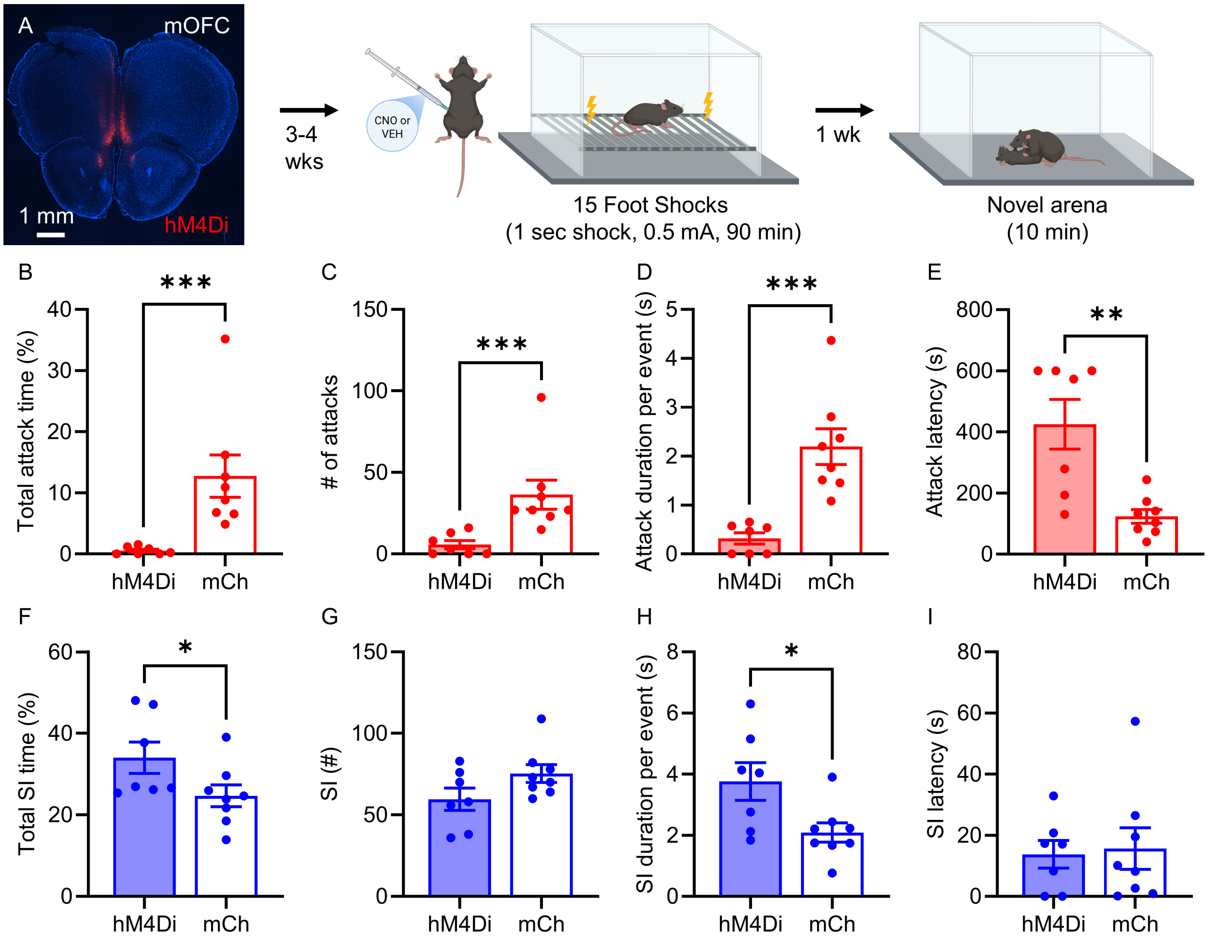


**Supplementary Fig. 8. Inhibiting excitatory mOFC neurons suppresses traumatic stress-induced aggression and non-aggressive social behavior.** (A) Experimental schedule and representative image of mice injected with AAV-CaMKIIα-hM4Di or mCherry (mCh) control virus into the mOFC (Bregma 2.34 mm), followed 3 to 4 weeks later by IP injections of 2 mg/kg CNO 30 min before receiving the foot shocks. (B-I) Quantification of aggressive (B-E) or non-aggressive social (F-I) behavior from mice expressing hM4Di or mCherry control and treated with CNO 30 min before receiving foot shocks (n = 7, 8). Mean +/- SEM. *p<0.05, **p<0.01, ***p<0.001.

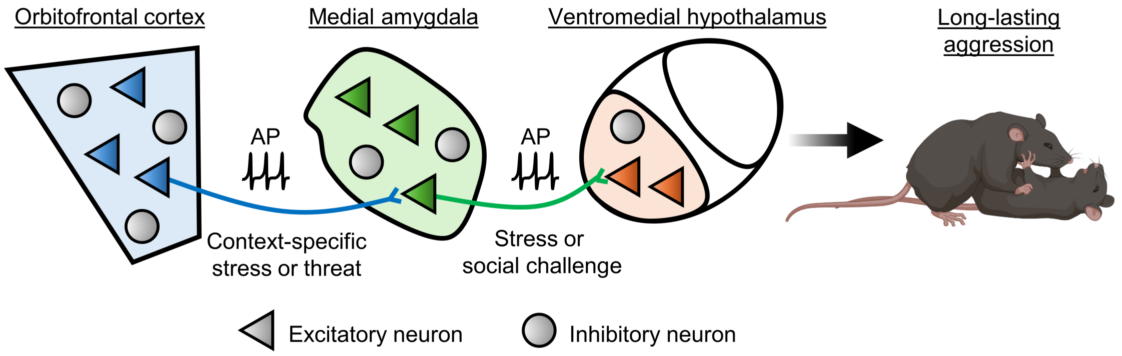


**Supplementary Fig. 9. Hypothesis for how the excitatory mOFC-MeApv-VmHvl pathway mediates traumatic stress-induced aggression.** Traumatic stress may hijack normal OFC function, leading to hyperactivation of the MeApv that potentiates the excitatory MeApv-VmHvl synapses. These changes could then lead to an overevaluation of threat, producing context-insensitive excessive aggression during a socially challenging situation.
